# Effects of Treatment on Structural and Functional Parameters of the Left Heart in Naïve Acromegaly Patients: Prospective Single-Centre Study: 12-Month Follow-Up

**DOI:** 10.3390/jcm14103397

**Published:** 2025-05-13

**Authors:** Ivana Ságová, Tomáš Bolek, Milan Dragula, Martin Jozef Péč, Jakub Benko, Jakub Jurica, Ingrid Tonhajzerová, Daniela Kantárová, Marián Mokáň, Peter Vaňuga, Matej Samoš

**Affiliations:** 1 Department of Endocrinology, National Institute of Endocrinology and Diabetology, 034 91 Lubochna, Slovakia; 2 1st Department of Internal Medicine, Jessenius Faculty of Medicine in Martin, University Hospital Martin, Comenius University in Bratislava, 036 59 Martin, Slovakia; 3 Department of Physiology, Jessenius Faculty of Medicine in Martin, Comenius University in Bratislava, 036 01 Martin, Slovakia

**Keywords:** acromegaly, body composition parameters, dual-energy X-ray absorptiometry, echocardiography, insulin-like growth factor 1, left heart

## Abstract

**Background/Objectives:** Cardiovascular diseases are the most prevalent comorbidities in patients with acromegaly (APs). Acromegalic cardiomyopathy is the leading cause of mortality in APs. This study aimed to assess changes in morphology and function of the left heart in naïve APs 12 months after the beginning of acromegaly treatment and to explore the effects of disease activity and body composition parameters on changes in the left heart. **Methods:** This prospective study involved 34 APs and 34 healthy controls (CON) matched for age, gender, and BMI. DXA and 2D echocardiography were performed at diagnosis and 12 months after the beginning of the treatment. **Results:** In APs, the prevalence of left ventricular (LV) hypertrophy was 70%. LV mass index (LVMI) was greater in APs compared to CON (124 vs. 86 ± g/m^2^, *p* < 0.001), but with no difference in size and systolic function of the LV. APs presented with increased left atrium volume (LAVI) and with diastolic dysfunction of the LV. Twelve months after the beginning of acromegaly treatment, IGF-1 levels decreased significantly (*p* < 0.001), and biochemical control of disease was achieved in 73.52% of APs. We found that in all APs, LAVI and LVMI decreased (all *p* < 0.05), and diastolic function of the LV improved without changes in systolic function. In multiple analyses, the changes in body surface area (β = −0.444, *p* < 0.001) and in lean body mass (β = −0.298, *p* = 0.027) were independent predictors of reverse remodelling of LVMI after the treatment. **Conclusions:** This study confirmed remodelling reversal of the left heart structure, followed by an improvement in diastolic function in naïve APs 12 months after the beginning of acromegaly treatment.

## 1. Introduction

Acromegaly is a rare disease characterized by excess secretion of growth hormone (GH) and insulin-like growth factor 1 (IGF-1), which in approximately 99% of cases is caused by a benign GH-secreting pituitary adenoma [1]. A meta-analysis of 16 studies confirmed an overall 72% increase in mortality in patients with acromegaly compared to the general population [2]. Cardiovascular (CV) diseases are the most prevalent comorbidities in patients with acromegaly, as well as the leading cause of death [3,4]. The most common CV complications are arterial hypertension (AH), acromegalic cardiomyopathy (ACM), coronary artery disease, valvular disease, and arrhythmia [5]. ACM is characterized by left ventricle hypertrophy and diastolic dysfunction, which can progress to impaired systolic function [6]. Echocardiography is the most widely used non-invasive, readily accessible technique for confirming the presence and severity of the mentioned structural and functional cardiac abnormalities [7]. The mechanism of the development of cardiac abnormalities in acromegaly has not been fully understood yet. The risk factors for developing cardiac abnormalities in acromegaly are prolonged hypersecretion of GH/IGF-1, diagnostic delay, higher body mass index (BMI), and increasing age [8]. Other significant CV risk factors are concomitant diseases (diabetes mellitus, obesity, dyslipidaemia, sleep apnoea syndrome) that also contribute to decreasing quality and shortening of life in patients with acromegaly (APs) [8]. The presence of any CV disease in APs exhibits a twofold increase in mortality compared to APs without such complications [9]. Nowadays, mortality rates in acromegaly decrease due to increased use of neurosurgery, development of new effective treatments, and increased use of radiosurgery in the last several years [10]. Recent studies have confirmed that strict control of GH/IGF-1 levels reduces mortality risk [11,12,13]. After effective treatment, cardiac abnormalities in acromegaly are reversible to a normal state, especially in young patients with short disease duration and GH/IGF-1 levels under control [14,15,16]. These abnormalities rarely improved in APs with uncontrolled GH/IGF-1 levels [17]. Acromegaly develops slowly, which causes delayed diagnosis, usually 8–10 years after onset [18]. Therefore, the early diagnosis and treatment of acromegaly is necessary to prevent CV impairment [19].

In the present study, we investigated structural and functional parameters of the left-sided heart chambers using 2D echocardiography in newly diagnosed APs compared with a matched healthy control group (CON) at baseline. We determined changes in these parameters in APs 12 months after the beginning of acromegaly treatment to test the hypothesis that cardiac impairment in APs is reversible after successful treatment of acromegaly. Furthermore, we aimed to investigate the interrelationships between left atrial and ventricular morphology, left ventricular function, body composition parameters, disease activity, and glucose metabolism in order to identify potential risk factors for cardiac impairment in acromegaly.

## 2. Materials and Methods

### 2.1. Patients and Controls

This single-centre prospective study was performed from September 2017 to April 2024 at the National Institute of Endocrinology and Diabetology in Ľubochňa and was approved by the regional medical ethics committee. Informed consent was obtained from all subjects.

The patient group consisted of 34 newly diagnosed APs (19 females and 15 males).

The inclusion criteria for APs were as follows:

- Diagnosis of acromegaly: GH > 1 µg/l during 75 g oral glucose tolerance test before receiving any treatments, increased IGF-1 levels, and positive pituitary magnetic resonance imaging findings [20]
- No history of acromegaly treatment.

The control group comprised 34 healthy subjects (19 females and 15 males) matched for age, sex, and BMI, and without the presence of acromegaly (normal IGF-1 levels).
The exclusion criteria for both groups were as follows:

- History of coronary artery disease, myocardial infarction or stroke, Grade II to IV valve disease severity, history of pulmonary embolism, LVEF < 50%, peripheral arterial disease, chronic renal failure, or chronic obstructive pulmonary disease.

Due to exclusion criteria, we excluded 4 APs.

All enrolled patients underwent baseline assessments, including physical examination, laboratory tests, 2D echocardiography, and dual-energy X-ray absorptiometry (DXA) at the time of acromegaly diagnosis. These evaluations were repeated 12 months following the initiation of treatment, which consisted of either transsphenoidal surgery (TSS) or somatostatin analogue (SSA) therapy. TSS was performed on 26 APs, 10 of whom received preoperative SSA therapy (lanreotide autogel 120 mg subcutaneously every 4 weeks). Postoperatively, 17 patients achieved remission, while 9 required continued SSA therapy.

The remaining 8 patients were primarily treated with SSA, and this treatment continues. In 2 of them, co-treatment with GH receptor antagonist (Pegvisomat 10 mg once a day s.c.) was added. No patients underwent additional surgery or radiotherapy during the study period. Endocrine remission was defined as a random GH < 1 µg/l or a nadir GH < 0.4 µg/l during oGTT and normal IGF-1 values for age and sex. After 12 months of treatment, 25 out of 34 patients had achieved adequate control of acromegaly.

### 2.2. Clinical Examination

Anthropometric measurements: height (cm), weight (kg), waist circumference (cm), BMI, and body surface area (BSA) were obtained for all study participants. BMI was calculated as weight (kg) divided by height squared (m^2^), and BSA was calculated using Mosteller’s formula [21]. Arterial hypertension (AH) was diagnosed according to the latest criteria established by the European Society of Hypertension [22]. The presumed duration of acromegaly was estimated by comparing old photographs and the onset of clinical symptoms/signs of acromegaly.

### 2.3. Laboratory Examinations

Blood samples were obtained between 07:00 and 08:00 a.m. after an 8 h fasting period. Pituitary hormones, their target hormones, creatinine, liver enzymes, lipid profile, serum fasting glucose, insulin, and glycated haemoglobin were measured using standardized laboratory methods. DM or prediabetes was diagnosed based on the recent criteria [23]. Insulin resistance (IR) was assessed using the homeostasis model assessment (HOMA IR), calculated using the following formula: fasting insulin (µU/L) x fasting glucose (mmol/L)/22.5 [24]. To measure IGF-1/GH levels, chemiluminescent immunometric assays ECLIA (Siemens Healthcare Diagnostics Products Ltd., Camberley, UK) were used. The intra-assay coefficient of variation (CV) for IGF-1 ranged from 3.0% to 7.6%, and for GH from 6.5% to 6.6%. The normal reference range for IGF-1 was adjusted for sex and age.

### 2.4. Echocardiography

Two-dimensional transthoracic echocardiography (2D TTE) was performed using a GE Vivid E9 ultrasound machine (GE Healthcare, Horten, Norway) by the same experienced investigator. All participants underwent 2D TTE at baseline (APs before the initial treatment of acromegaly) and APs again 1 year following the beginning of the treatment. Standard echocardiographic views were used for all measurements. The left atrium (LA) and left ventricle (LV) dimensions were obtained in accordance with the European Society of Echocardiography recommendations [25]. LV end-diastolic diameter as well as myocardial thickness—interventricular septum (IVS) and posterior wall (PW)—were measured. Left ventricular mass (LVM) was calculated using the Devereux formula, and LVM was indexed to BSA to obtain the left ventricular mass index (LVMI). LV hypertrophy (LVH) was defined as LVMI >115 g/m^2^ for men or >95 g/m^2^ for women. Relative wall thickness (RWT) was calculated using the following formula: 2x PW end-diastolic diameter (PWDd)/LV end-diastolic diameter (LVEDd) [26]. Concentric LVH was defined as increased LVM and RWT > 0.42 cm, while eccentric LVH was defined as increased LVM and RWT < 0.42 cm. LV ejection fraction (EF) was calculated using the Simpson biplane method, where a low normal level was set at EF = 55% according to the current guidelines. LA volume index (LAVI) was measured using the area–length method in both the 4-chamber and 2-chamber apical views. LV diastolic function was assessed in the 4-chamber apical view by measuring peak velocities of early (E) and late (A) diastolic flow, as well as pulsed early diastolic waves (E′), and calculating the E/A and E/E′ ratios using tissue Doppler imaging. Diastolic dysfunction was graded from I to III using the current guidelines [27]. Right ventricular (RV) diameters were measured in the 4-chamber apical view, and tricuspid annular plane systolic excursion (TAPSE) was assessed using M-mode echocardiography to measure the peak excursion of the lateral tricuspid annulus.

### 2.5. Dual-Energy X-Ray Absorptiometry (DXA)

Body composition parameters were measured using DXA (Hologic Horizon A, Bedford, MA, USA) with 13.6 version software. The coefficient of variation for fat mass was 0.78% and for lean mass 0.52%. The analysis included total body fat mass, trunk fat mass, limb fat mass, total body lean mass, trunk lean mass, and limb lean mass. DXA was performed in all participants at baseline and in APs once more 12 months after the beginning of acromegaly treatment.

### 2.6. Statistical Analyses

All statistical analyses were performed using IBM SPSS version 25 (IBM SPSS Statistics, IBM Corporation, Chicago, IL, USA). Continuous variables are presented as medians (interquartile range), and categorical variables as numbers and percentages. To compare continuous values between AP and CON, the Mann–Whitney U test was used. Categorical variables were compared using the chi-square test. The Wilcoxon signed-rank test was used to compare continuous values between baseline and 12 months after examinations. Correlation analyses were performed using the Spearman correlation coefficient. The change in each variable was denoted by Δ, equal to the pre-treatment value minus the posttreatment value. Predictors of baseline LVMI and LVMI reduction were calculated using univariate analyses and multiple regressions (stepwise method) as covariate factors. Statistical significance in all statistical tests was defined as a *p*-value less than 0.05.

## 3. Results

### 3.1. Characterization of the Study Population at the Time of Diagnosis

The baseline characteristics of all study participants are detailed in Table 1. The median age of 34 enrolled newly diagnosed APs (19 F, 15 M) was 52 (IQR 41.5–59). The median age of 34 CON (19 F, 15 M) was 53 (IQR 40.8–61).

### 3.2. Comparison Between APs and CON

No difference in age was present between APs and CON. The APs had a higher prevalence of AH compared to CON (46.7 vs. 18%, *p* < 0.001). There was no statistically significant difference in arterial blood pressure (BP) between both groups, and their BP was well controlled using antihypertensive drugs (Table 1). Dyslipidaemia was predominantly present in the APs compared to CON (66.7% vs. 33%, *p* < 0.001) (Table 1). There was no significant difference in prevalence of DM between the groups, but prevalence of prediabetes was higher in the APs compared to CON (32% vs. 7%, *p* < 0.001). Serum levels of GH, IGF-1, and IGF-1/ULN were significantly higher in the APs than in CON (*p* < 0.001) (Table 1). We did not find significant differences in BMI, BSA, or fat mass (total, trunk, limb) between both groups, but lean mass (total, trunk, limb) was higher in the APs compared to CON (all *p*  <  0.05) (Table 1).

In measured echocardiography parameters, we found no differences in the size of the right and left ventricles between both groups, but LVEDd tended to be higher in the APs (50 vs. 49 mm, *p* = 0.079). Compared to CON, the APs presented with increased LVMI (124 vs. 86 ± g/m^2^, *p* < 0.001) (Table 2). The prevalence of LVH was 70% in the APs (86.7% concentric/13.3% eccentric), while in CON, it was 23%. Significant differences between APs and CON were confirmed in LAVI (38.4 vs. 28.7 mL/m^2^, *p* < 0.001). There were no statistically significant differences in systolic function between both groups, but we found differences in diastolic function measured with E/É and E/A (Table 2).

### 3.3. Comparison Between Male and Female APs

No significant differences were observed in the prevalence of AH, prediabetes/DM, and dyslipidaemia between male and female APs (Table 1). However, male APs exhibited higher levels of IGF-1 (IGF/ULN) and glycated haemoglobin compared to females. Statistically significant differences in body composition parameters were noted between the genders. Male patients had greater height, weight, WC, BMI, BSA, and lean body mass (total, trunk, and limbs) (Table 1). In contrast, female patients had a higher fat mass (total, trunk, limbs). Furthermore, male APs showed increased LAVI, LVEDd, IVSd, PWDd, and LVM/LVMI, with no significant differences in the diastolic and systolic function of LV compared to females.

### 3.4. Changes in Biochemical, Body Composition, and Echocardiography Parameters (12 Months After the Beginning of Acromegaly Treatment)

In all APs (in both sexes), the follow-up GH, IGF-1 levels, and IGF-1/ULN decreased (all *p*  <  0.05) (Table 3). Biochemical control of acromegaly was achieved in 73.52% of APs. There was no change in BMI or BSA, but the fat mass increased and lean mass decreased 12 months after treatment (Table 3). Echocardiographic evaluations demonstrated that 12 months after the beginning of acromegaly treatment, LAVI decreased in all APs in both genders (all *p*  <  0.05), without significant change in LVEDd. A significant reduction in LVMI was observed 12 months after treatment (124 g/m^2^ (97–173) vs. 111 g/m^2^ (83–154), *p* < 0.001). The median (25th and 75th percentiles) change from baseline for LVMI was −11 (−14; −6) in females and −20 (−24; −6) in males. We also found improvement in diastolic function measured with E/É and E/A (in all AP and females). We did not find improvement in systolic function of the LV, but the females reached borderline statistical significance (*p* = 0.053) (Table 3).

### 3.5. Relationships Between Activity of Acromegaly, Body Composition Parameters, and Echocardiography Parameters in APs at Baseline

**Figure 1:**
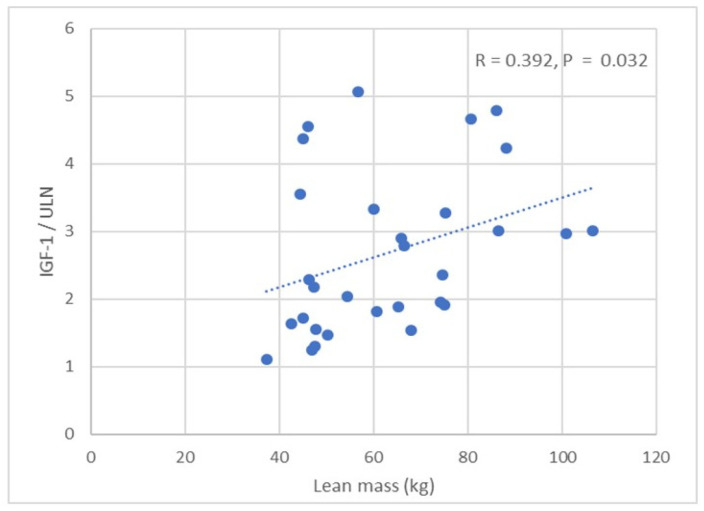
Correlation between IGF-1/ULN and lean mass in patients with acromegaly.

No significant correlation was observed between IGF-1 and BMI. IGF-1 was positively correlated with BSA (R = 0.472, *p* = 0.006). No correlation was observed between IGF-1 and fat mass. IGF-1 was positively correlated with lean mass in all APs (R = 0.392, *p* = 0.032) (Table 4) (Figure 1), including males and females (*p* < 0.05). We did not find any correlations between age, BP, and structural/functional parameters of the left heart. IGF-1, BMI, BSA, and lean mass (total, trunk, limb) positively correlated with LAVI (all *p* < 0.05) (Table 4). We did not find any correlations between glycated haemoglobin, fat mass, and LAVI. BMI, BSA, lean mass (total, trunk, limb), and glycated haemoglobin were positively correlated with LVEDd (*p* < 0.05).

**Figure 2:**
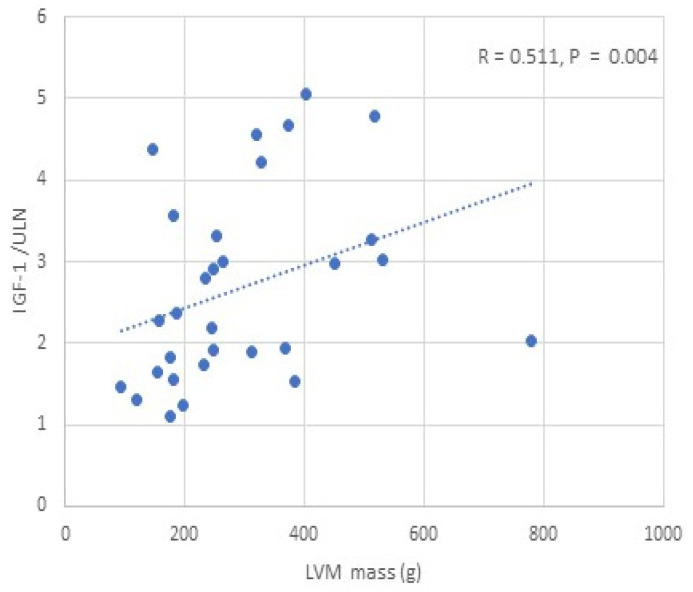
Correlation between IGF-1/ULN and LVM in patients with acromegaly.

**Figure 3:**
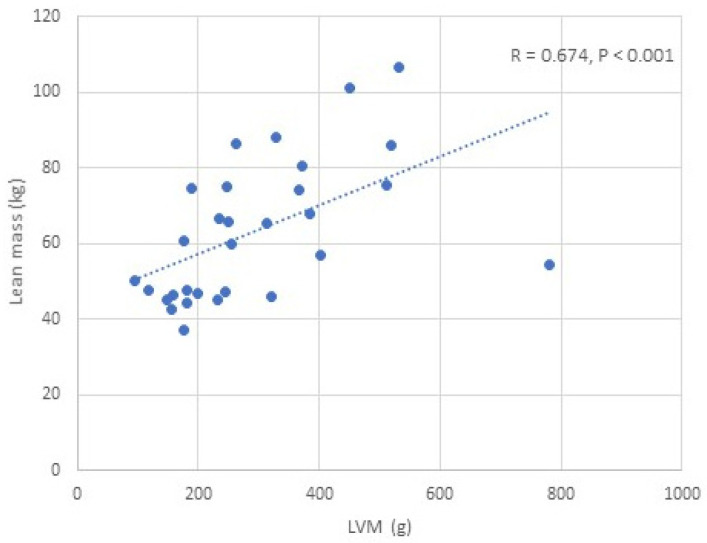
Correlation between lean mass and LVM in patients with acromegaly.

We found a positive correlation between IGF-1 and LVM/LVMI, while glycated haemoglobin was negatively correlated with LVM/LVMI in all APs, males and females (*p* < 0.05) (Table 4) (Figure 2). BMI, BSA, and lean mass (total, trunk, limb) were positively correlated with LVM/LVMI in all APs, males and females (*p* < 0.05) (Table 4, Figure 3). No significant correlation was observed between fat mass and LVM/LVMI. We did not find any correlations between the activity of acromegaly, body composition parameters, glycated haemoglobin, and LV diastolic function. BMI, BSA, and lean/fat mass were positively correlated with LVEF (*p* < 0.05) (Table 4). No correlations were observed between IGF-1 and LVEF.

### 3.6. Predictors of Baseline LVMI and Predictors of Reverse Remodelling of LVMI

Body composition parameters, disease activity parameters, parameters of glucose metabolism, and echocardiographic parameters were analysed to identify the predictors of baseline LVMI. In the univariate analysis, baseline IGF-1, BMI, BSA, HbA1c, lean mass, and LVEDd appeared to be predictors of baseline LVMI. However, in the multiple analysis, the best predictors of baseline LVMI were baseline LVEDd (β = 1.11, *p* < 0.001) and baseline lean mass (β = 0.385, *p* < 0.001) (Table 5). 

We also analysed predictors of LVMI regression 12 months after the beginning of acromegaly treatment. In the univariate analysis, Δ BSA, Δ lean mass, Δ IVSDd, and Δ LVEDd appeared to predict the reduction in LVMI (all *p* < 0.05). In the multiple analyses, the Δ body surface area (β = −0.444, *p* < 0.001) and Δ lean mass (β = −0.298, *p* = 0.027) were independent predictors of reverse remodelling of LVMI (Table 6).

### 3.7. Association Between LVM and Body Composition Scaling Parameters

In the univariate analysis, LVM showed the strongest correlation with measured lean mass (R = 0.674, *p* < 0.001). Of all the clinically derived scaling parameters, the weakest correlation was found between LVM and height (R = 0.450, *p* = 0.013). A stronger correlation was observed between LVM and BSA (R = 0.591, *p* = 0.001). Correlation between LVM and fat mass reached only a borderline significant trend (R = 0.323, *p* = 0.082). In the multiple analyses, out of all parameters, lean mass was the only clinically derived parameter that was independently correlated with LVM (β = 0.420, *p* < 0.001).

## 4. Discussion

In this prospective study, we examined pre-treatment structural and functional parameters of the left heart chambers using 2D echocardiography, and body composition characteristics using DXA in naïve patients with acromegaly. Subsequently, 12 months after the beginning of acromegaly treatment, we analysed the reversibility of cardiac impairment and the changes in body composition.

There is a wide spectrum of cardiovascular comorbidities in acromegaly, ranging from AH to acromegalic cardiomyopathy. The presence of any of them at the time of diagnosis indicates a significant increase in the risk of death [17]. It is estimated that 20–78% of APs will develop LVH [5,14,15,28]. In clinical practice, 2D TTE is the most widely used technique for assessing LVM that is typically normalized to BSA, often referred to as LVMI. In acromegaly, disease activity and duration, AH, age, and BMI play a significant role in the development of LVH [5]. Several autopsy studies have reported higher prevalence of LVH, myocardial fibrosis, and infarction in APs [29,30]. The increased prevalence of LVH and impaired diastolic function in APs has also been confirmed by some clinical studies using 2D TTE [5,28,31]. Recent studies have rarely described progression to overt systolic dysfunction in APs with uncontrolled disease (< 3% of patients) [31,32,33].

The exact prevalence of different cardiac abnormalities in acromegaly remains unclear and is still a topic of study. Our study found a higher prevalence of LA enlargement, LVH, and LV diastolic dysfunction (Grade I in 87.9%) in APs compared to controls, but no difference in size and systolic function of LV. The prevalence of LVH in APs was 70%, predominantly concentric in 86.7%, and LVM/LVMI were significantly greater in APs compared to CON. These findings are concordant with those of Gadelha P. et al. [7], Popielarz-Grygalewicz A. et al. [30], as well as our previous research (cross-sectional study on 129 APs) [5]. In contrast, other studies have reported a low prevalence of LVH in acromegaly [34,35].

GH and IGF-1 are very important regulators of body composition and cardiometabolic risk. Their overproduction leads to increased myocyte contractility, deposition of collagen, disruption of muscle fibres, and lymphocyte infiltration within the myocardium, which can eventually progress to developing acromegalic CMP. Excess GH/IGF-1 can also disrupt local water balance, leading to increased myocardial water content and the development of cardiac oedema [17]. Body composition is also impacted. Excess GH/IGF-1 has an anabolic effect on skeletal muscle and a lipolytic effect on adipose tissue, and it also affects total body water retention [36]. In this study, the baseline characteristics of body composition obtained before the treatment were as follows. The lean mass (total, trunk, limb) was higher in all APs (both sexes) compared to CON. IGF-1 positively correlated with lean mass in all APs (both sexes). Interestingly, sex differences apparently influenced the changes in fat mass in the APs. There were no differences in fat mass (total, trunk, limb) between all APs compared to CON, but total fat decreased in males but not in females. The less pronounced changes in female APs could be explained by the effect of oestrogen, which can suppress GH conversion to IGF-1 in the liver and then slow down lipolysis by inhibiting the fat oxidation process [37].

Studies have confirmed that successful acromegaly treatment tends to increase fat mass, while lean mass decreases, which can be attributed to changes in metabolic processes due to declining GH/IGF-1 levels [38,39,40]. In our study, 12 months after the beginning of acromegaly treatment, IGF-1 levels decreased significantly (*p* < 0.001) and biochemical control of disease was achieved in 73.52% of APs. Biochemical control did not change BMI or BSA in the short term but led to a less favourable phenotype in APs with increased fat mass and decreased lean mass in both sexes. We found no significant correlation between fat mass and IGF-1. Our results suggest that the increases in fat mass are offset by the losses in lean mass. The reduction in skeletal muscle mass may account for the clinical experience of fatigue reported by many APs during the postoperative period. In their study, Fuchtbauer et al. addressed this issue, showing that proximal muscle fatigue and grip strength increased after achieving biochemical remission in APs [41].

In our study, simultaneously with the decrease in disease activity and changes in body composition after the treatment, the APs also exhibited changes in the structural and functional parameters of the left heart. We confirmed the beneficial impact of acromegaly treatment on some of the structural parameters of the left heart. There was a significant decrease of LVM/LVMI in the APs. The correlation found between the LVM/LVMI and IGF-1 highlights the significant effect of IGF-1 in LVH pathogenesis in acromegaly. Our findings are consistent with several previous studies that demonstrated that both surgical intervention and 12 months of first-line SSA therapy significantly reduced LVM as well as improved LVH [42,43,44,45,46]. However, other studies have not confirmed this association [34,35]. The precise role of IGF-1 in the pathophysiology of LVM in acromegaly is still not fully understood. We also found improvement in diastolic function of the LV, which we assess as secondary to the reduction of LVM.

We identified several clinically relevant predictors. The baseline LVMI was significantly influenced by IGF-1, BMI, BSA, lean mass, and glycated haemoglobin. In the multiple analyses, only lean body mass was identified as an independent factor that significantly influenced baseline LVMI. This provides a clue that increased LVMI could be associated with increased skeletal muscle in acromegaly. The larger heart structures of patients with a greater lean mass may be caused by the increased demand for oxygen and nutrients by the muscles, leading to compensatory cardiac adaptations. Among the variables that are used to calculate LVMI, we found that only LVEDd was an independent predictor of baseline LVMI, and it showed a positive relationship.

We also examined predictors of LVMI regression following 12 months of acromegaly treatment. In the univariate analysis, changes in BSA, lean mass, IVS, and LVEDd were identified as potential predictors of a reduction in LVMI. In the multivariate analysis, only changes in BSA and changes in lean mass were identified as independent predictors of the reverse remodelling of LVMI. In the multiple analyses, out of the body composition scaling parameters (height, BSA, fat mass, lean mass), only lean mass was identified as a clinical parameter independently correlating with LVM. Therefore, our results confirm that lean body mass measured using dual-energy X-ray absorptiometry strongly correlates with LVM in acromegaly, and it suggests that lean body mass could be the ideal scaling variable for LVM in acromegaly.

We had hypothesized that there would be differences in the prevalence of cardiac abnormalities between males and females with acromegaly. We found that male APs exhibited higher levels of IGF-1 compared to females. Furthermore, male APs showed increased LAVI, LVEDd, IVSd, PWDd, and LVM/LVMI. There were no significant differences in the diastolic and systolic function of the LV between the sexes. In both sexes, we also identified a correlation between body composition parameters and structural cardiac abnormalities. Based on these findings, we suppose that cardiac parameters are affected by the different sizes of the body and muscle mass in males and females. It is unclear to what extent the differences in left heart impairment between the sexes could also be influenced by the differences in elevated IGF-1 levels, which, as mentioned above, could lead to increased LVM, suggesting its role in cardiac hypertrophy in APs. Further investigation is necessary to establish this.

At baseline, we found increased LAVI in APs compared to CON. Increased LAVI in acromegaly could be a direct consequence of LVH and myocardial fibrosis, or of LV diastolic dysfunction, or if it is a result of elevated GH receptor expression in LA cardiomyocytes. Other studies have also found impaired morphology of LA in acromegaly, including increased LAVI and LA anteroposterior diameter [5,7,30,31]. Recently, a study by Uziȩbło-Życzkowska et al. reported significant LA dysfunction across all phases of its function: as a reservoir, conduit, and booster pump in APs [31]. In our study, 12 months of acromegaly treatment led to decreased LAVI in both sexes. Before treatment, LAVI positively correlated with IGF-1 and lean mass, i.e., with the parameters that later significantly decreased due to the treatment. Our results suggest that the decrease in LAVI could be caused by the postoperative rapid reduction of GH/IGF-1, leading to a reduction in lean mass and a subsequent reduction in LVH. The correlation between LAVI and IGF-1 suggests that IGF-1 has a meaningful impact on LA enlargement in APs.

To the best of our knowledge, this is the first prospective study in treatment-naïve APs that examines the effects of acromegaly treatment on changes in structural and functional parameters of the left heart, as well as on body composition, while also evaluating their interrelationships. The key finding of our study is that changes in lean mass, which are directly influenced by GH/IGF-1 overproduction, could play an important role in the pathogenesis of LVH and in the enlargement of LA in acromegaly. The treatment resulted not only in a reduction of GH/IGF-1 levels but also in decreased lean mass, a significant reduction in LVM and LAVI, and improved diastolic function. Our results demonstrate a strong association between lean mass and LVM in acromegaly, suggesting that lean mass could serve as an ideal scaling variable for LVM in the detection of LVH in this condition.

## 5. Conclusions

In conclusion, our study confirmed a higher prevalence of LA enlargement, LV diastolic dysfunction, and LVH in treatment-naïve patients with acromegaly compared to the control group. Twelve months after the beginning of acromegaly treatment, there was a significant reduction in LVM and LAVI, with improved diastolic function. These changes strongly correlated with decreases in both IGF-1 levels and lean mass. Thus, early and appropriate treatment of acromegaly can improve cardiac impairment in patients suffering from this condition.

### Strengths and Limitations

The strengths of this study include its prospective design with follow-up and the use of two different methodologies, 2D echocardiography and DXA, which allowed for a comprehensive analysis and comparison of multiple factors within a single cohort. However, there are several limitations. It is a single-centre study with a relatively short follow-up period and a relatively small sample size, attributable to the low incidence of acromegaly, and with gender disparities in patient recruitment. Other important echocardiographic measures, such as strain analysis, were not assessed, as they were not available in the first years of this study. Acromegaly treatment was not the same for all the patients. They were treated with transsphenoidal surgery and/or SSA. This could have influenced the results, as some studies have demonstrated that SSA treatment may offer greater benefits in reversing CMP compared to surgery, indicating a potential direct positive effect of SSA on cardiomyocytes [47]. Given these limitations, further studies will be required to confirm our results.

## Figures and Tables

**Table 1:** Baseline characteristics of all study subjects.

Characteristics	Patients with Acromegaly (APs) (n = 34)	Control Group (CON) (n = 34)	Comparison APs vs. CON *p*-Value	Male APs (n = 15)	Female APs (n = 19)	Comparison Male vs. Female APs *p*-Value
Sex (M/F)	15/19	15/19	-	-	-	-
Age at the time of study (year)	52 [41.5–59]	53 [40.8–61]	NS	49.7 [37–53.8]	53.5 [46–59.3]	0.043
Smoking history (%)	19%	17%	NS	23%	13%	NS
Arterial hypertension (%)	46.7%	18%	<0.001	40.7%	47.6%	NS
Systolic BP (mmHg)	123.6 [115.7–140]	122.0 [110–137]	NS	122.7 [114–139]	123.9 [116.5–141]	NS
Diastolic BP (mmHg)	78.3 [66.5–89.2]	77.1 [64.7–87.9]	NS	80.2 [73–92.1]	78.6 [66–90.7]	NS
Diabetes (%)	3.3%	0%	NS	0%	5.6%	NS
Prediabetes (%)	32%	7%	<0.001	29%	35%	NS
Dyslipidaemia (%)	66.7%	33%	<0.001	60.3%	72.2%	NS
**Laboratory examinations**						
Baseline GH (ng/mL)	6.5 [5.2–6.1]	0.2 [0.1–0.3]	<0.001	10.5 [6.7–12.6]	4.4 [2.9–7]	0.002
IGF-1 (ng/mL)	526 [359–710]	139 [109–165]	<0.001	654 [525–942]	417 [323–605]	0.029
IGF1/ULN	2.3 [1.7–3.4]	0.6 [0.5–0.7]	<0.001	3 [2.1–4.6]	2 [1.5–3]	0.023
Creatinine (µmol/l)	65.9 [2.8–11.1]	73.5 [61.8–87]	0.033	71.8 [65.7–89.9]	57.2 [51.3–73]	0.007
FPG (mmol/L)	5.8 [5.2–6.1]	5.4 [4.7–5.6]	0.012	5.5 [5.2–5.9]	5.7 [5.1–6.2]	NS
HOMA IR	3.1 [2.2–4.5]	1.7 [1.1–3.3]	0.002	3.4 [2.2–4.6]	2.9 [2.2–4.2]	NS
Glycated haemoglobin (%)	5.6 [5.3–5.8]	5.2 [5.0–5.5]	0.004	5.7 [5.4–5.9]	5.4 [5.3–5.8]	0.032
Total cholesterol (mmol/L)	5.2 [4.7–5.6]	5.1 [4.2–5.6]	NS	5.3 [4.4–5.7]	4.9 [4–5.5]	NS
LDL cholesterol (mmol/L)	3.5 [2.9–4.4]	3 [2.2–3.4]	0.008	3.6 [2.2–4.5]	2.9 [2.2–3.2]	0.035
HDL cholesterol (mmol/L)	1.3 [1.1–1.8]	1.4 [1.2–1.7]	NS	1.4 [1.1–1.9]	1.3 [1.1–1.8]	NS
Triglycerides (mmol/L)	1.2 [1.0–1.6]	1.35 [1.2–1.9]	NS	1.2 [1.1–1.5]	1.2 [1.0–1.8]	NS
**Anthropometric measurements**						
Height (cm)	173 [166–181]	170 [164–175]	NS	180 [175–189]	165 [161–176]	<0.001
Weight (kg)	90 [73–110]	88 [69–106]	NS	109 [85–118]	78 [69–94]	<0.001
Waist circumference (cm)	100 [89–116]	102 [83–106]	NS	107 [95–119]	94 [86–109]	0.003
BMI (kg/m^2^)	31 [25.0–34.1]	30.5 [24.1–33.9]	NS	33 [29–36]	29 [24–34]	<0.001
BSA (m^2^)	2 [1.81–2.23]	1.99 [1.75–2.18]	NS	2.28 [2.04–2.46]	2 [1.78–2.01]	<0.001
**DXA measurements**						
Fat mass total (kg)	30.1 [22.8–37.5]	31.3 [25.1–37.8]	NS	29 [21.9–36.8]	32.3 [23.8–37.8]	0.019
Fat mass trunk (kg)	15 [10.1–17.2]	15.2 [11.8–19,6]	NS	14.6 [9.7–17.7]	15.9 [10.1–17.2]	0.048
Fat mass limb (kg)	14.8 [11.6–17.5]	14.1 [11.6–18.5]	NS	13.2 [9.1–16.2]	15.1 [12.6–19.2]	0.026
Lean mass total (kg)	60.4 [46.8–75]	49.1 [42.5–66.7]	0.033	77.8 [64.1–81.8]	50.5 [45–65.3]	<0.001
Lean mass trunk (kg)	30.9 [24.6–38.1]	26.8 [22.8–34]	0.048	40 [31.8–44.8]	26.9 [24.2–33.4]	<0.001
Lean mass limb (kg)	27.7 [20.4–35.6]	21.5 [18.9–31.2]	0.041	36.2 [31.7–42]	23 [19.5–28.1]	<0.001

Data are presented as median (standard interquartile range) and as percentage; level of significance was set at *p* ≤ 0.05. BP: blood pressure, GH: growth hormone, BMI: body mass index, BSA: body surface area, DXA: dual-energy X-ray absorptiometry, FPG: fasting plasma glucose, HDL: high-density lipoprotein, HOMA IR: homeostasis model assessment of insulin resistance, IGF-1: insulin-like-growth factor 1, LDL: low-density lipoprotein, ULN: upper limit normal.

**Table 2:** Echocardiography parameters.

Characteristics	Patients with Acromegaly (APs) (n = 34)	Control Group (CON) (n = 34)	Comparison APs vs. CON *p*-Value	Male APs	Female APs	Comparison Male vs. Female APs *p*-Value *
Ao. Asc. (mm)	34 [32–35]	33 [31–34]	NS	34 [32.3–35]	33 [31.8–35]	NS
LAVI (mL/m^2^)	38.4 [35–41]	28.7 [24–31]	<0.001	38.6 [35–42]	36.9 [33.9–38]	0.048
LVEDd (mm)	50 [46.8–56]	49 [46–50]	0.079	53 [50–61]	48 [44.8–55.3]	<0.001
RVEDd (mm)	32 [29.6–35]	31 [29–33.7]	NS	33.5 [30.4–36]	30 [29.1–33.7]	0.036
IVSDd (mm)	13 [11–15]	10 [9–11]	<0.001	14 [13–16]	12 [9.8–13.3]	<0.001
PWDd (mm)	12 [11–15]	10 [9–10]	<0.001	13 [12–15]	12 [10–13]	0.027
LVM (g)	248 [180–375]	167 [142–196]	<0.001	348 [249–438]	215 [158–315]	<0.001
LVMI (g/m^2^)	124 [97–173]	86 [74–100]	<0.001	153 [107–190]	116 [90–160]	<0.001
LVH (%)	70%	23%	<0.001	72%	67%	NS
RWT > 0.42 (%)	86.7%	13.3%	<0.001	91.7%	83.3%	NS
LVEF (%)	60 [55–60]	56 [55–60]	NS	58.5 [55–60]	60 [55.8–60.5]	NS
E/É	11.0 [9–12]	9.3 [8.6–10]	0.033	11.2 [9–12.2]	10.6 [8.8–11]	NS
E/A	0.9 [0.6–1.2]	1.16 [0.7–1.6]	0.027	0.87 [0.7–1.1]	0.9 [0.75–1.2]	NS
TAPSE	23 (21; 24)	23 (23; 24)	NS	23 [20.5–23.8]	23.5 [20–24.5]	NS

Data are presented as median (standard interquartile range) and as percentage. The level of significance was set at *p* ≤ 0.05. Ao. Asc.: ascending aorta, LAVI: left atrial volume index, LVEDd: left ventricular end-diastolic parameter, RVEDd: right ventricular end-diastolic parameter, IVSDd: interventricular septum diastolic diameter, PWDd: posterior wall diastolic diameter, LVM: left ventricular mass, LVMI: left ventricular mass index, LVH: left ventricle hypertrophy, RWT: relative wall thickness, LVEF: left ventricular ejection fraction, E/É: mitral E-wave velocity divided by mitral annular velocity, E/A: mitral E-wave divided by mitral A- wave velocity, TAPSE: tricuspid annular plane systolic excursion.

**Table 3:** Comparison of biochemical characteristics, body composition parameters, and echocardiographic parameters in APs at baseline and 12 months after the beginning of acromegaly treatment.

Characteristics	At Diagnosis (All APs)	1-Year Follow-Up (All APs)	*p*-Value (All APs)	1-Year Follow-Up Change (Female APs)	*p*-Value (Female)	1-Year Follow-Up Change (Male APs)	*p*-Value (Male)
IGF-1 (ng/mL)	526 [359–710]	212 [167–269]	<0.001	−211 (−378; −130)	<0.001	−332 (−592; −317)	0.002
IGF-1/ULN	2.3 [1.7–3.4]	0.99 [0.71–1.25]	<0.001	−1.05 (−1.87; −0.61)	<0.001	−2.02 (−2.94; −1.2)	0.003
BMI (kg/m^2^)	31.8 [25.0–34.1]	30.7 [26.0–34.0]	NS	0 (−1; 1)	NS	0 (−2; 1)	NS
BSA (m^2^)	2.00 [1.81–2.23]	1.99 [1.83–2.24]	NS	−0.01 (−0.04; 0.02)	NS	0 (−0.05; 0.02)	NS
Fat mass (kg)	30.1 [22.8–37.5]	31.2 [24.6–39.4]	0.035	1.0 (0.2; 2.8)	0.048	2.1 (0.3; 5.1)	0.030
Fat mass trunk (kg)	15 [10.1–17.2]	15.9 [10.5–17.4]	0.040	0.4 (0; 1.8)	0.057	0.8 (0.2; 2.2)	0.036
Fat mass limb (kg)	14.8 [11.6–17.5]	15 [11.7–17.6]	NS	0 (0; 0.5)	NS	0 (0.1; 0.9)	NS
Lean mass (kg)	60.4 [46.8–75]	56.1 [45.9–72]	0.010	−1.5 (−3.2; −0.4)	0.010	−3.5 (−7.4; −1.2)	0.018
Lean mass trunk (kg)	30.9 [24.6–38.1]	28.8 [23.1–36.9]	0.006	−1.2 (−2.7; −0.2)	0.008	−2.4 (−5.3; −1)	0.012
Lean mass limb (kg)	27.7 [20.4–35.6]	26.4 [19.3–35]	0.031	−0.6 (−1.2; −0.1)	0.025	−1.5 (−3.2; −0.7)	0.039
LAVI (ml/m^2^)	38.4 [35–41]	36.2 [34.1–39.7]	0.027	−0.7 (−1; 0)	0.026	−1.2 (−1.6; 0)	0.032
LVEDd (mm)	50 [46.8–56]	49 [46.5–56]	NS	−0.4 (−0.8; 0)	NS	−0.5 (−1.8; 0)	NS
IVSd (mm)	13 [11–15]	11 [10–14]	<0.001	−1 (−1; −0,8)	<0.001	−1 (−2; −1)	0.004
PWDd (mm)	12 [11–15]	12 [10–14.5]	<0.001	−0.2 (−1; 0)	0.017	−1 (−1; 0)	0.008
LVM (g)	248 [180–375]	217 [163–328]	<0.001	−21 (−30; −11)	<0.001	−44 (−66; −18)	0.003
LVMI (g/m^2^)	124 [97–173]	111 [83–154]	<0.001	−11 (−14; −6)	<0.001	−20 (−24; −6)	0.004
LVEF (%)	60 [55–60]	59 [55–60]	NS	0 (0; 1)	NS	−0.5 (0; 2)	0.053
E/É	11.0 [9–12]	10.7 [8.8–11.6]	0.037	−0.2 (−0.4; 0.3)	0.031	0 (0; 0)	NS
E/A	0.9 [0.6–1.2]	0.9 [0.4–1.3]	0.047	0.15 (−0.15; 0.1)	0.039	0 (−0.02; −0.05)	NS

Data are presented as median (standard interquartile range). Level of significance was set at *p* ≤ 0.05. APs: patients with acromegaly, GH: growth hormone, IGF-1: insulin-like-growth factor 1, BMI: body mass index, BSA: body surface area, IVSd: interventricular septum diastolic diameter, LAVI: left atrial volume index, LVEDd: left ventricular end-diastolic parameter, LVM: left ventricular mass, LVMI: left ventricular mass index, LVEF: left ventricular ejection fraction, E/É: mitral E-wave velocity divided by mitral annular velocity, E/A: mitral E-wave divided by mitral A-wave velocity, PWDd: posterior wall diastolic diameter, ULN: upper limit normal.

**Table 4:** Correlation analyses in Aps.

Characteristics	IGF-1 (ng/mL)	IGF1/ULN	BMI (kg/m^2^)	BSA (m^2^)	Lean Mass (kg)	Lean Mass Trunk (kg)	Lean Mass Limb (kg)	Fat Mass (kg)	Glycated Haemoglobin (%)
**LAVI (mL/m^2^)**	R = 0.313 *p* = 0.045	R = 0.385 *p* = 0.034	R = 0.432 *p* = 0.025	R = 0.621 *p* < 0.001	R = 0.424 *p* = 0.027	R = 0.432 *p* = 0.017	R = 0.4382 *p* = 0.037	NS	NS
**LVEDd (mm)**	NS	NS	R = 0.549 *p* < 0.001	R = 0.599 *p* < 0.001	R = 0.624 *p* < 0.001	R = 0.701 *p* < 0.001	R = 0.553 *p* = 0.002	NS	R = 0.369 *p* = 0.045
**LVM (g)**	R = 0.463 *p* = 0.016	R = 0.511 *p* = 0.004	R = 0.532 *p* = 0.002	R = 0.591 *p* = 0.001	R = 0.674 *p* < 0.001	R = 0.689 *p* < 0.001	R = 0.593 *p* < 0.001	R = 0.323 *p* = 0.082	R = −0.473 *p* = 0.008
**LVMI (g/m^2^)**	R = 0.394 *p* = 0.031	R = 0.489 *p* = 0.006	R = 0.395 *p* = 0.031	R = 0.399 *p* = 0.029	R = 0.451 *p* = 0.012	R = 0.422 *p* = 0.020	R = 0.328 *p* = 0.080	NS	R = −0.371 *p* = 0.043
**LVEF (%)**	NS	NS	R = 0.394 *p* = 0.032	R = 0.421 *p* = 0.020	R = 0.459 *p* = 0.011	R = 0.464 *p* = 0.010	R = 0.457 *p* = 0.011	R = 0.364 *p* = 0.048	NS
**E/É**	NS	NS	NS	NS	R = −0.374 *p* = 0.055	NS	NS	NS	NS
**E/A**	NS	NS	NS	NS	NS	R = 0.397 *p* = 0.033	NS	NS	NS

Level of significance was set at *p* ≤ 0.05, R–Spearman correlation coefficient. APs: patients with acromegaly, GH: growth hormone, IGF-1: insulin-like-growth factor 1, BMI: body mass index, BSA: body surface area, LVEDd: left ventricular end-diastolic parameter, LVM: left ventricular mass, LVMI: left ventricular mass index, LVEF: left ventricular ejection fraction, E/É: mitral E-wave velocity divided by mitral annular velocity, E/A: mitral E-wave divided by mitral A-wave velocity.

**Table 5:** Univariate and multiple analyses evaluating the main predictor of baseline LVMI in patients with acromegaly.

Baseline Variable	Univariate Analysis		Multiple Analysis		
	ρ	*p*-Value	B	β	*p*-Value
**IGF-1**	0.394	**0.031**	11.194	0.200	0.525
**HBA1c (%)**	0.371	**0.043**	27.846	0.118	0.258
**BMI (kg/m^2^)**	0.395	**0.031**	−2.747	−0.240	0.551
**BSA (m^2^)**	0.381	**0.038**	−52.865	−0.229	0.697
**Lean mass (kg)**	0.438	**0.016**	−1.385	−0.385	**<0.001**
**LVEDd (mm)**	0.875	**<0.001**	9.850	1.11	**<0.001**

Level of significance was set at *p* ≤ 0.05. BMI: body mass index, B: unstandardized B, β: standardized coefficient β, BSA: body surface area, HBA1c: glycated haemoglobin, IGF-1: insulin-like-growth factor 1, LVEDd: left ventricular end-diastolic parameter, ULN: upper limit normal.

**Table 6:** Univariate and multiple analyses of LVMI reduction predictors 12 months after the beginning of acromegaly treatment.

	Univariate Analysis		Multiple Analysis		
Variable	ρ	*p*-Value	B	β	*p*-Value
Δ **IGF-1**	0.218	0.342			
Δ **HBA1c**	0.122	0.598			
Δ **BMI**	0.392	0.072			
Δ **BSA**	0.453	**0.026**	−127.313	−0.444	**<0.001**
Δ **Lean mass**	0.484	**0.018**	−3.081	−0.298	**0.027**
Δ **LVEDd**	0.469	**0.032**			
Δ **IVSD**	0.436	**0.046**			
Δ **PWDd**	0.335	0.138			

Level of significance was set at *p* ≤ 0.05; Δ: change 12 months after the beginning of acromegaly treatment, BMI: body mass index, B: unstandardized B, β: standardized coefficient β, BSA: body surface area, HBA1c: glycated haemoglobin, IGF-1: insulin-like-growth factor 1, IVSDd: interventricular septum diastolic diameter, LVEDd: left ventricular end-diastolic parameter, LVMI: left ventricular mass index, PWDd: posterior wall diastolic diameter.

## Data Availability

The original contributions presented in this study are included in the article. Further inquiries can be directed to the corresponding author.
